# Transient Radial-Dominant Neuropathy Following the Use of Sterile Exsanguination Tourniquet During the Posterior Plating of a Humeral Shaft Fracture: A Case Report

**DOI:** 10.7759/cureus.113731

**Published:** 2026-07-31

**Authors:** Kamil Balaban

**Affiliations:** 1 Orthopedics and Traumatology, Ministry of Health Finike State Hospital, Antalya, TUR

**Keywords:** humeral shaft fracture, radial nerve palsy, silicone ring tourniquet, sterile exsanguination tourniquet, tourniquet paralysis

## Abstract

Tourniquet-related nerve injury is an uncommon but clinically important complication of extremity surgery. Postoperative radial nerve palsy after humeral shaft fixation is usually attributed to fracture-related injury or surgical manipulation, particularly when a posterior approach is used. We present a case of transient radial-dominant neuropathy following the use of a sterile exsanguination tourniquet during the posterior plating of a distal humeral shaft fracture. A 27-year-old male construction worker with an AO/OTA (Arbeitsgemeinschaft für Osteosynthesefragen/Orthopaedic Trauma Association) 12A1 spiral fracture of the distal humeral shaft underwent open reduction and internal fixation through a posterior triceps-splitting approach. A sterile exsanguination tourniquet was applied distal to the axilla and proximal to the incision and fracture line for 70 minutes. The radial nerve was identified, looped, and protected intraoperatively. The patient had a normal preoperative neurological examination but developed early postoperative wrist drop, dorsal hand numbness, and palmar-digital paresthesia. Four-week nerve conduction studies demonstrated severe proximal radial neuropathy with milder proximal median nerve involvement and relative preservation of ulnar motor conduction. At four months, repeat studies showed marked electrophysiological recovery, and the patient achieved complete clinical recovery by one year. The radial-dominant but median-associated proximal conduction pattern, together with the location of tourniquet application and recovery profile, was compatible with a compressive neurapraxic mechanism rather than isolated surgical radial nerve trauma alone. Sterile exsanguination tourniquet-related neuropathy should be considered in the differential diagnosis of postoperative radial nerve palsy after humeral shaft fixation, especially when neurological findings extend beyond an isolated radial nerve distribution.

## Introduction

Tourniquets are widely used in extremity surgery to provide a bloodless operative field and improve surgical visualization. Although generally considered safe, tourniquet-related nerve injury (TRNI) remains a rare but clinically important complication. The proposed mechanisms include mechanical compression, pressure gradients at the cuff or ring margins, focal demyelination, conduction block, and, in more severe cases, axonal injury [1-3].

Sterile elastic exsanguination tourniquets, including silicone ring-based systems, combine limb exsanguination and arterial occlusion in a single sterile device. Their advantages include rapid application, maintenance of a wide sterile field, and effective bloodless exposure, particularly in upper-extremity procedures requiring proximal access [4-6]. However, because these devices apply circumferential compression through a relatively narrow ring, focal nerve compression remains a theoretical and clinically relevant concern [3,4]. In the available TRNI literature, reported upper-extremity cases have predominantly involved pneumatic tourniquets, whereas detailed electrophysiological documentation of nerve injury following the use of sterile elastic exsanguination tourniquet remains limited [1,7]. At the proximal upper arm, such circumferential compression may affect more than one nerve and may therefore mimic an isolated surgical mononeuropathy.

Postoperative radial nerve palsy after humeral shaft fixation is usually attributed to fracture-related injury or surgical manipulation, especially when a posterior approach is used [8]. However, when neurological findings extend beyond an isolated radial nerve distribution, an alternative mechanism such as proximal circumferential compression should be considered. We present a case of transient radial-dominant neuropathy with mild median nerve involvement following the use of a sterile exsanguination tourniquet during the posterior plating of a humeral shaft fracture, despite intraoperative identification and protection of the radial nerve.

## Case presentation

A 27-year-old male construction worker with no previous medical history or prior surgery presented to the emergency department after a fall from height. Physical examination revealed pain, swelling, and functional limitation of the affected arm. There were no associated injuries. Preoperative neurovascular examination was normal, with intact radial, median, and ulnar nerve motor and sensory function. In particular, wrist extension, finger extension, thumb extension, thumb opposition, intrinsic hand function, and sensation over the dorsal hand, palm, and fingers were preserved.

**Figure 1:**
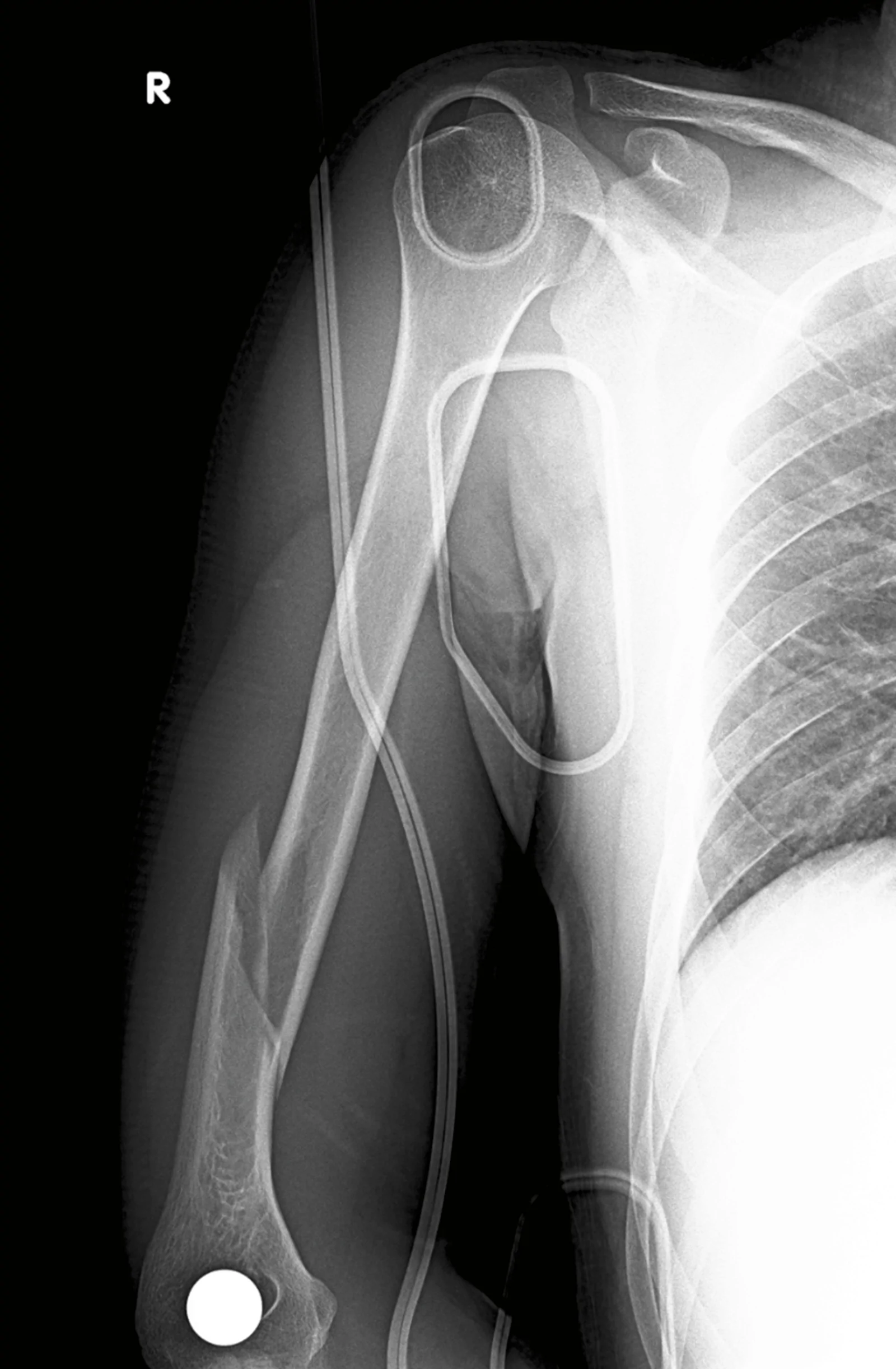
Preoperative radiograph of the humeral shaft fracture. Preoperative anteroposterior radiograph demonstrating an AO/OTA 12A1 spiral fracture of the distal humeral shaft. AO/OTA: Arbeitsgemeinschaft für Osteosynthesefragen/Orthopaedic Trauma Association.

Plain radiographs demonstrated a spiral fracture of the distal humeral shaft, classified as AO/OTA (Arbeitsgemeinschaft für Osteosynthesefragen/Orthopaedic Trauma Association) 12A1 (Figure 1). The patient was scheduled for open reduction and internal fixation.

**Figure 2:**
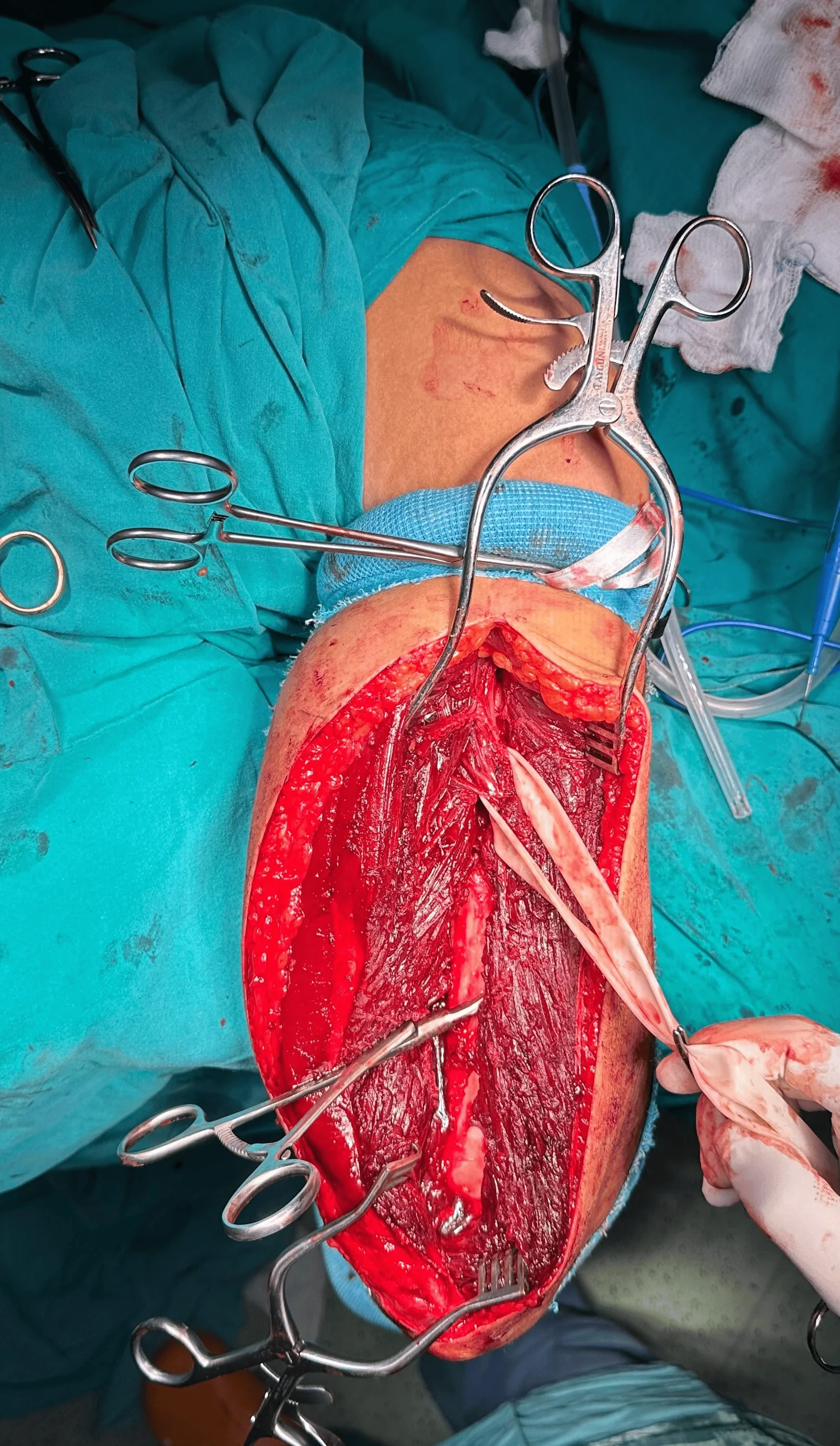
Intraoperative view after sterile exsanguination tourniquet application. Intraoperative photograph showing the sterile exsanguination tourniquet applied proximally, distal to the axilla. The radial nerve is identified and looped, and the distal humeral shaft fracture is temporarily held with a reduction clamp.

Surgery was performed under general anesthesia with the patient in the lateral decubitus position. Before surgical exposure, a sterile exsanguination tourniquet was applied distal to the axilla, proximal to both the planned incision and the fracture line. A posterior triceps-splitting approach was used. The radial nerve was identified intraoperatively, looped, and protected throughout the procedure. No macroscopic nerve discontinuity, entrapment, crushing, or compression was observed (Figure 2).

**Figure 3:**
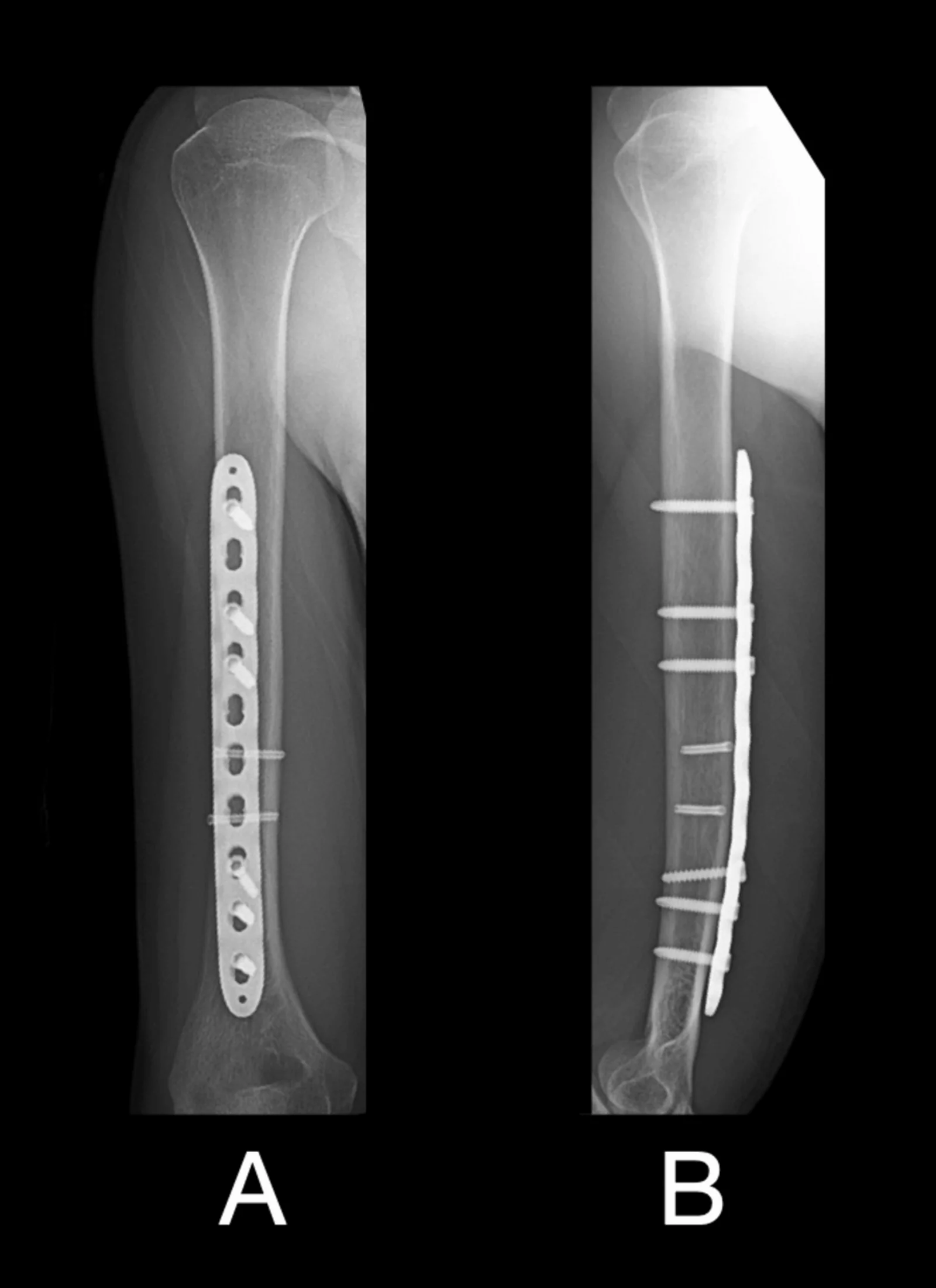
Postoperative radiographs after posterior plate fixation. Postoperative anteroposterior and lateral radiographs demonstrating posterior plate fixation with satisfactory fracture reduction and implant position.

The fracture was reduced and fixed with a posterior plate. The total tourniquet application time was 70 minutes. Postoperative anteroposterior and lateral radiographs showed satisfactory reduction and implant position (Figure 3).

In the early postoperative period, the patient developed wrist drop, numbness over the dorsum of the hand, and paresthesia involving the palm and fingers. These findings were consistent with predominant radial nerve dysfunction, with additional symptoms suggestive of median nerve involvement. Ulnar nerve-related motor findings were not prominent. There was no clinical evidence of compartment syndrome, expanding hematoma, vascular compromise, wound complication, or implant malposition. Conservative management was initiated immediately after recognition of the neurological deficit, including observation, wrist splinting, and rehabilitation.

At four weeks postoperatively, because of persistent neurological symptoms, nerve conduction studies were performed (Table 1). The study demonstrated predominant radial nerve involvement, with milder median nerve abnormalities and relative preservation of ulnar motor conduction. Radial motor amplitude decreased from 2.6 mV with forearm stimulation to 0.7 mV with axillary stimulation, accompanied by proximal conduction slowing to 37.0-37.5 m/s. These findings were consistent with severe proximal radial neuropathy, suggesting focal demyelination, partial conduction block, and possible axonal involvement. Median motor conduction showed milder proximal involvement, with amplitude reduction from 6.4 mV at the wrist to 3.2 mV at the axilla and slowing of the elbow-axilla segment to 35.7 m/s. Ulnar motor conduction was relatively preserved.

**Table 1:** Serial electrophysiological findings of radial, median, and ulnar nerve function

Nerve	Four-week findings	Four-month findings	Interpretation
Radial nerve	Motor amplitude decreased from 2.6 mV with forearm stimulation to 0.7 mV with axillary stimulation; proximal conduction velocity slowed to 37.0-37.5 m/s	Motor amplitudes improved to 5.9-6.8 mV; conduction velocities improved to 52.6-58.3 m/s	Severe proximal radial neuropathy at four weeks with marked electrophysiological recovery by four months
Median nerve	Motor amplitude decreased from 6.4 mV at the wrist to 3.2 mV at the axilla; elbow-axilla conduction velocity slowed to 35.7 m/s	Motor amplitudes improved to 11.0-11.6 mV; conduction velocities normalized to 55.6-56.1 m/s	Mild proximal median nerve involvement with subsequent normalization
Ulnar nerve	Motor conduction relatively preserved	Motor conduction remained preserved	No significant ulnar motor involvement

Conservative treatment was continued, and progressive neurological recovery was observed during follow-up. At six weeks, the sensory symptoms had substantially regressed, and wrist and finger extension had improved to Medical Research Council grade 4/5. Complete motor and sensory recovery was observed at approximately two months. At the four-month electrophysiological assessment, nerve conduction studies demonstrated marked recovery. Median motor amplitudes improved to 11.0-11.6 mV, and conduction velocities normalized to 55.6-56.1 m/s. Radial motor amplitudes improved to 5.9-6.8 mV, and radial conduction velocities increased to 52.6-58.3 m/s. These findings supported a recovering compressive neuropathy, predominantly affecting the radial nerve and, to a lesser extent, the median nerve. The pattern was most compatible with a primarily neurapraxic lesion with focal demyelination and partial conduction block, although limited axonal involvement could not be excluded because needle electromyography was not performed.

**Figure 4:**
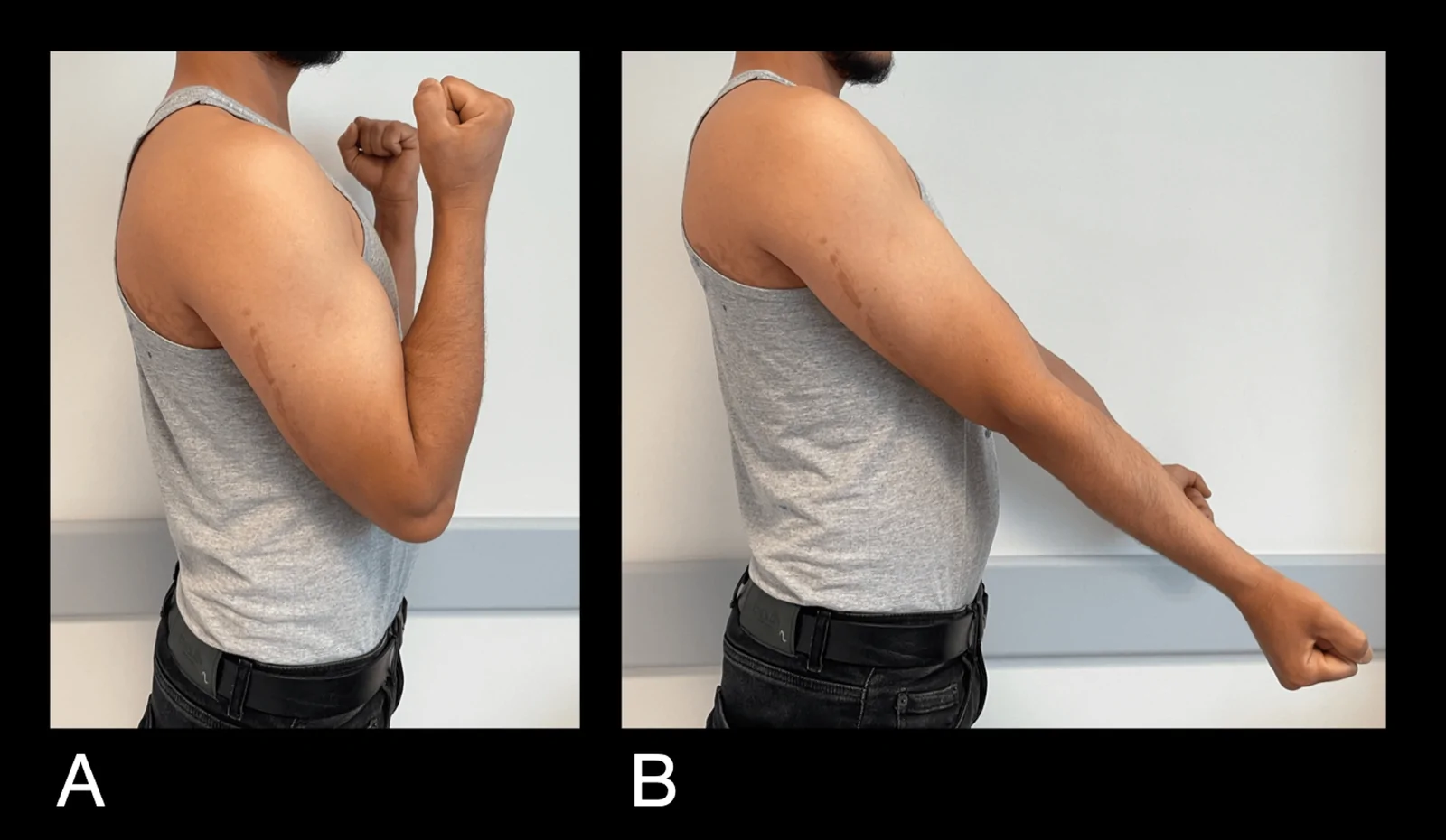
One-year clinical follow-up. Clinical photographs at the one-year follow-up demonstrating full elbow range of motion and normal functional recovery.

At the one-year follow-up, recovery was maintained, with no residual motor or sensory deficits and full elbow range of motion (Figure 4).

## Discussion

Postoperative radial nerve palsy after humeral shaft fixation is commonly attributed to fracture-related injury or surgical manipulation, particularly when a posterior approach is used. This explanation remains possible in the present case because the radial nerve was exposed and looped during posterior plating. However, careful intraoperative identification of the nerve does not fully explain the accompanying proximal median nerve abnormality.

The electrophysiological pattern was therefore central to the interpretation of this case. The initial nerve conduction study demonstrated severe proximal radial neuropathy, but the lesion was not an isolated radial mononeuropathy. Mild proximal median nerve involvement was also present, whereas ulnar motor conduction was relatively preserved. Clinically, this corresponded to wrist drop and dorsal hand numbness, together with palmar and digital paresthesia. Because the median nerve was outside the posterior surgical field, this radial-dominant but multi-nerve pattern supports a proximal circumferential compression mechanism rather than isolated surgical radial nerve trauma alone. The proximal conduction slowing and amplitude reduction, followed by marked recovery, were most consistent with focal demyelination and partial conduction block rather than a fixed structural nerve injury.

Tourniquet-related nerve injury is strongly linked to focal compression and pressure gradients. Experimental and clinical studies have shown that conduction delay and block occur at the level of the tourniquet, especially near the border zones, where mechanical stress and pressure gradients are greatest [2,3]. Theoretically, a narrow sterile elastic ring may concentrate pressure over a smaller surface area and produce rapid local soft tissue deformation. Drosos et al. reported that silicone ring tourniquet application may cause early pain and grip strength reduction because the narrow ring applies pressure over a small area, although median nerve conduction impairment was greater with pneumatic tourniquet application in their volunteer study [5]. Similarly, Kovar et al. did not demonstrate greater nerve compression with the silicon ring tourniquet compared with a pneumatic tourniquet in short-term MRI-based volunteer testing [9]. Therefore, the available evidence does not prove that sterile elastic ring tourniquets are intrinsically more neurotoxic than pneumatic cuffs; rather, nerve risk appears to depend on local compression characteristics, pressure distribution, duration, limb contour, application level, and proximity of nerves to bone [3,5,9].

Most previously reported upper-extremity tourniquet palsy cases have involved pneumatic tourniquets [1,7,10]. In contrast, sterile elastic exsanguination or silicone ring tourniquets are less frequently represented in detailed neurological case reports. Published clinical series of sterile ring tourniquets generally report low complication rates, and neurological complications appear uncommon [4-6]. For example, in a hand surgery series using the sterile silicon ring self-exsanguinating tourniquet, one neuropraxia was reported and resolved by six months [6]. Bourquelot and Levy also noted rare reported paresis events with elastic ring tourniquets and emphasized that nerve complications are related to compression force and duration, although much of the available safety data is observational or based on unpublished manufacturer-related reports [4]. Therefore, the present case adds clinically relevant electrophysiological detail to a relatively underreported complication pattern.

An additional point is that the tourniquet time in this case was 70 minutes, which is not unusually prolonged. This is important because tourniquet-related nerve injury has been reported over a wide range of application times and pressures, and no universally safe threshold has been established [11,12]. Therefore, the absence of excessive duration does not exclude a tourniquet-related mechanism. In a narrow circumferential device, local pressure distribution, ring position, limb contour, and proximity of superficial nerves to bone may be as relevant as total application time [3,4].

Alternative explanations must be considered. A posterior humeral approach can cause radial nerve neuropraxia despite direct visualization. Positioning-related brachial plexus traction, regional anesthesia-related neuropathy, postoperative hematoma, implant-related irritation, pre-existing neuropathy, and cervical radiculopathy are also important differential diagnoses in postoperative upper-extremity nerve deficits [8,13]. In this patient, preoperative neurological examination was normal, surgery was performed under general anesthesia without regional block, there was no evidence of hematoma, vascular compromise, implant malposition, compartment syndrome, or pre-existing neuropathy, and the neurological abnormality included a proximal median component. These findings do not prove causality, but they strengthen the hypothesis of a sterile exsanguination tourniquet-related compressive neuropathy.

This case has limitations. First, it is a single observation, and causality cannot be definitively established. Second, needle electromyography was not performed, limiting the direct assessment of active denervation, Wallerian degeneration, and reinnervation; therefore, limited axonal involvement could not be excluded. Third, exact local tissue pressure beneath the sterile exsanguination tourniquet could not be measured. Finally, no validated functional outcome measure was recorded; clinical recovery was assessed using serial neurological examinations, nerve conduction studies, and range-of-motion assessment. Despite these limitations, the radial-dominant but multi-nerve proximal conduction pattern, the anatomical location of the tourniquet proximal to the surgical field, and the recovery profile collectively support sterile exsanguination tourniquet-related compression neuropathy as a credible mechanism.

The practical implication is that postoperative wrist drop after humeral shaft fixation should not automatically be attributed to surgical radial nerve injury when a proximal sterile exsanguination tourniquet has been used. Although this complication appears rare, surgeons should be aware that sterile exsanguination tourniquet use in upper-arm surgery may be followed by radial-dominant compressive neuropathy. Preoperative counseling should also include the possibility of postoperative nerve dysfunction, including transient neurapraxia, even when the nerve is carefully identified and protected and no direct intraoperative nerve injury is observed. Careful patient selection, correct sizing and placement, avoidance of unnecessary proximal application, minimizing tourniquet time, and postoperative assessment of radial, median, and ulnar nerve function are advisable. When neurological deficit occurs, nerve conduction studies should evaluate not only the radial nerve but also the median and ulnar nerves, as additional proximal nerve abnormalities may help distinguish circumferential tourniquet-related compression from isolated surgical radial neuropraxia.

## Conclusions

Sterile exsanguination tourniquet-related neuropathy should be considered in the differential diagnosis of postoperative radial nerve palsy after humeral shaft fixation, particularly when the neurological findings are not limited to the radial nerve distribution. In this case, the radial-dominant but median-associated proximal conduction abnormality, the anatomical location of tourniquet application proximal to the surgical field, and the subsequent clinical and electrophysiological recovery were compatible with a compressive neurapraxic mechanism. Although causality cannot be definitively proven from a single case, this report highlights that rare nerve complications may occur even with relatively short sterile exsanguination tourniquet application times and careful intraoperative radial nerve protection.
